# Mycelium-Based Composites as a Sustainable Solution for Waste Management and Circular Economy

**DOI:** 10.3390/ma17020404

**Published:** 2024-01-13

**Authors:** Daniel-Gabriel Barta, Irina Simion, Ancuța-Elena Tiuc, Ovidiu Vasile

**Affiliations:** 1 B&G Family Innovation SRL, Street Tăutului 242B, 407280 Cluj-Napoca, Romania; gabriel.barta@rongodesign.com (D.-G.B.); simion.fa.irina@student.utcluj.ro (I.S.); 2 Faculty of Materials and Environmental Engineering, Technical University of Cluj-Napoca, Muncii Boulevard 103-105, 400641 Cluj-Napoca, Romania; 3 Department of Mechanics, Faculty of Biotechnical Systems Engineering, National University of Science and Technology Politehnica Bucharest, Splaiul Independentei 313, 060042 Bucharest, Romania; ovidiu_vasile2002@yahoo.co.uk

**Keywords:** mycelium-based composite, circular economy, acoustic properties, natural adhesive, spent coffee grounds, agricultural waste

## Abstract

The global population is expected to increase by nearly 2 billion individuals over the next three decades, leading to a significant surge in waste generation and environmental challenges. To mitigate these challenges, there is a need to develop sustainable solutions that can effectively manage waste generation and promote a circular economy. Mycelium-based composites (MBCs) are being developed for various applications, including packaging, architectural designs, sound absorption, and insulation. MBCs are made by combining fungal mycelium with organic substrates, using the mycelium as a natural adhesive. Mycelium, the vegetative part of fungi, can be grown on various organic feedstocks and functionalized into a range of diverse material types that are biobased and thus more sustainable in their production, use, and recycling. This work aims to obtain mycelium-based composites with acoustic absorption properties, using coffee grounds and agricultural waste as raw materials. The topic approached presents a new method of recovering spent coffee grounds that does not involve high production costs and reduces two current environmental problems: noise pollution and abundant waste. Measurements of the normal-incidence sound absorption coefficient were presented and analyzed. Mycelium-based composites offer an innovative, sustainable approach to developing bio-composite sound-absorbing surfaces for interior fittings. The material by *Ganoderma lucidum* exhibits exceptional sound-absorbing properties at frequencies below 700 Hz, which is a crucial aspect of creating sound-absorbing materials that effectively absorb low-frequency sound waves. The modular construction system allows for a high degree of flexibility to adapt to short-term changes in the workplace.

## 1. Introduction

Significant population growth is a global challenge, escalating from the current 8 billion to approximately 9.7 billion by 2050 [1]. This rapid population growth necessitates advancements in technology to cater to the escalating needs of the populace, while simultaneously mitigating the potential adverse environmental impacts such as pollution, waste generation, and depletion of natural resources [2]. With the acceleration of population growth and urbanization, the annual generation of waste is projected to rise by approximately 70%. Specifically, global waste production, which was 2.01 billion tons in 2016, is expected to increase to 2.2 billion tons by 2025 and further to 3.40 billion tons by 2050 [3]. This increase in waste generation is a significant concern, as it not only poses environmental challenges but also places a substantial financial burden on municipal budgets [4]. Therefore, it is crucial to leverage technological advancements to develop sustainable solutions that can effectively manage the increasing waste generation and other environmental challenges associated with rapid population growth. Furthermore, these technological improvements should aim to promote a circular economy, thereby reducing our reliance on non-renewable resources and minimizing environmental pollution [5].

The current state of research on mycelium-based materials is promising, with studies exploring their potential as sustainable alternatives to traditional materials in various sectors. Mycelium, the vegetative part of fungi, can be grown on various organic feedstocks and functionalized into a range of diverse material types which are biobased and thus more sustainable in their production, use, and recycling [6].

Mycelium-based composites (MBCs) are innovative engineering materials that are being developed for various applications, including packaging, architectural designs, sound absorption, and insulation. These composites combine fungal mycelium with organic substrates, using the mycelium as a natural adhesive. The quality of the composite depends on the type of fungi and substrate used [1,7]. The multitude of existing international scientific studies on the development of mycelium-based materials using various wastes for their exceptional acoustic and thermal insulating properties is a confirmation of the choice of raw material, as well as certain factors that may affect the quality of the material such as nutritional content, availability and abundance, degradability, cost, compatibility, textural, and structural properties [7]. The species, substrate type, and manufacturing methods greatly influence the quality of mycelium-based materials. However, the effect of fungal species on final material properties is more dominant than the effect of substrate type [8].

Mycelium is the vegetative structure of fungi, consisting of a dense three-dimensional network of thin, tubular, branching filaments called hyphae. The hyphae grow by apical extension and branch repeatedly, allowing the mycelium to explore and invade the organic substrate on which the fungus is growing. As the hyphae grow, they secrete enzymes and acids that break down complex organic polymers such as cellulose, hemicellulose, lignin, and chitin into simpler organic monomers that can be absorbed as nutrients [9]. The secreted enzymes include cellulases, hemicelluloses, laccases, and chitinases [10]. The process of extracellular digestion and nutrient absorption is essential for fungal growth and reproduction. When two compatible monokaryotic mycelia meet and fuse, they form a dikaryotic mycelium that can undertake sexual reproduction and produce fruiting bodies such as mushrooms; as the mycelium ramifies through the substrate, the hyphae branch repeatedly and fuse to form a dense three-dimensional network [11]. This provides mechanical strength and rigidity to the mycelium, allowing it to bind and gradually colonize the organic particles into a continuous composite structure [10].

Nutrient substrates such as glucose serve as the main source of nutrients for fungi; to obtain this nutrient, some fungi break down cellulose into glucose. Substrates with a high cellulose content allow fungi to grow quickly; as a result, they correspond to a high tensile strength. This is actually due to the higher density of the mycelium and chitin content. However, some plant species such as hemp secrete a toxic substance that is incompatible with fungal growth. Such plant species should be selected to save the life of the mushroom. The best-known substrates for the production of mycelium-based materials are wood chips, sawdust, straw, coconut powder, garden waste, and baggage [12]. These substrates are selected because of their compatibility with fungal growth and their lignocellulosic fiber content, which is shown in Table 1. The growth rate of mycelium in straw substrate is faster than in sawdust; similarly, mycelium growth on baggage shows a faster growth rate than in sawdust and its mixture.

This is due to the nutritional variation and nutritional complexity of glucan in sawdust. In addition, straw and bagasse have softer particle properties than sawdust, so fungi can easily utilize nutrients from soft substrates rather than hard substrates. To increase the nutritional content, different supplements, such as wheat bran and rice bran, and different agricultural straws are mixed [13].

**Table 1:** Mycelium-based materials with different strains and substrates.

Fungal Species	Feedstocks/ Substrate	Sterilization Method	Inoculation Method	Packing Method	Temperature (°C)	Grow- Time	Dry Method	Application
*Trametes* *versicolor* [14]	Flax, flax dust, flax long treated fibers, flax long untreated fibers, flax waste, wheat straw dust, wheat	Autoclaved at 121 °C for 20 min	10% grain spawn	The 20% wt of fibers, 70% wt of sterile demineralized H_2_O, and 10% wt of mycelium; spawn was mixed and put in the PVC molds	28 °C	16 days	70 °C for 5 to 10 h	Thermal insulation
*Ganoderma lucidum* [15]	Sawdust wood	Not specified	Inoculated into molds	In polymer bag	25–35 °C	14 days	Heat processing above 70 °C, 5% moisture content	Foams/The core of sandwich structures
*Coriolus* (*Trametes*) *versicolor* and *Pleurotus* *ostreatus* [16]	Hemp hurd, wood chips, hemp mat, hemp fibers, non-woven mats	Boiling water for 100 min or 0.3% hydrogen-peroxide	10% or 20% pre-grown spawn cultivated on rye	Not specified	Room temperature	30 days	Oven at 125 °C and dry for 2 h	Foam
*Ganoderma lucidum* and *Pleurotus* *ostreatus* [17]	Cellulose and cellulose-PDB	Autoclaved at 120 °C for 15 min	Agar plug	Not specified	25–30 °C	20 days	Oven for 2 h at 60 °C	Fibrous film

Over the past decade, rapid urbanization and lifestyle changes have led to the pervasive presence of noise in people’s lives, resulting in the emergence of noise pollution as a significant environmental concern. Noise pollution is characterized by the propagation of unpleasant sounds in the environment over an extended period, which can have detrimental consequences for both humans and animals [18]. Sound absorption has become a critical factor in reducing noise pollution for human comfort. The high sound pressures generated by noise pollution from vehicles, industrial settings, and machinery have necessitated the development of cost-effective and efficient methods for producing sound-absorbing materials, known as sound absorbers [19].

The purpose of this article is to conduct theoretical and experimental investigations into the production and evaluation of sound-absorbing materials using agro-industrial waste as raw materials, as well as the mycelium of two distinct species of fungi functioning as binders. The elements of innovation within this research are represented by the following: (a) the identification of a new method of capitalizing the waste generated from coffee preparation that does not involve high production costs; (b) the use of coffee grounds as a raw material in the creation of materials with acoustic absorption properties is an important step in reducing the amount of waste, also having a nutritional role for the development of the mycelium network; (c) the use of the mycelium network as a natural binder, replacing resins and polyurethane foams usually used in obtaining sound-absorbing panels.

In the context of mycelium-based acoustic panels, the circular economy is relevant due to the use of waste materials such as coffee grounds and agricultural by-products as the substrate for mycelium growth. This approach aligns with the circular economy principles by repurposing waste into valuable products, reducing the consumption of virgin resources and minimizing environmental impact. Additionally, mycelium-based materials are biodegradable and recyclable, further contributing to the circular economy by closing the loop of resource utilization.

## 2. Materials and Methods

To obtain mycelium-based composites with acoustic absorption properties, we must make them a promising alternative to traditional materials in various sectors. Mycelium-based composites’ performance varies between samples depending on the substrate, fungal species used, and processing method.

To obtain the mycelium-based composites, the following composition was 90% of the substrate (spent coffee grounds, 20%; coffee chaff, 10%; hay straw, 30–40%; hemp dust, 15%; cereal mixture, 5%) and fungal species (10%) of *Ganoderma lucidum* (*G. lucidum*) and *Trametes versicolor* (*T. versicolor*).

The available literature in the field of mycelium-based composites is fragmented, leading to different methodologies and a lack of standardized and comparative presentation of production parameters. The production parameters of mycelium-based composites are influenced by factors such as the genetic nature of the species used, characteristics of the raw material, and variable parameters underlying the production process, which depend on specific laboratory conditions. These variable parameters include temperature during growth, humidity, access to oxygen, drying, and post-production methods [13].

The selection of the fungal species is a vital process in the manufacture of mycelium panels. The species chosen can have a significant impact on the characteristics of the final product. Commonly employed fungi for mycelium-based composites are white rot fungi, such as Pleurotus ostreatus and *Ganoderma lucidum* [17,20].

### 2.1. Substrate Preparation of Mycelium-Based Composites

**Figure 1:**
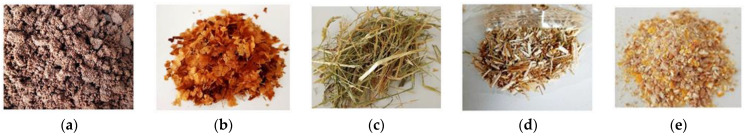
Substrate materials used for mycelium-based composites: (**a**) spent coffee grounds; (**b**) coffee chaff; (**c**) hay straw; (**d**) hemp dust; and (**e**) cereal mixture.

The raw materials had been ground to acquire a uniform distribution of grain sizes (2–4 mm), and the substrate had been supplied with a moisture content of 50–60% before being sterilized at 121 °C for 30 min to assure material composition decontamination. Images of substrate materials (spent coffee grounds, coffee chaff, hay straw, hemp dust, cereal mixture) used to prepare mycelium-based composites are presented in Figure 1.

The apparent density of the substrate materials selected for the mycelium-based composites, determined according to EN ISO 845:2009 [21], is presented in Table 2.

### 2.2. Inoculation

Coffee husk, hemp husk, cereal combination, and coffee chaff were inoculated with 10% spores of the fungal species *G. lucidum* and *T. versicolor*. Using 100 mm × 100 mm molds, a second layer of straw that was enriched with 10–20% moisture was spread out; moreover, the raw materials mixture was then pressured to a thickness of 40 mm and covered with a sterile foil.

### 2.3. Incubation

In the context of mycelium development, incubation refers to the period during which the mycelium grows in a dark environment at a temperature of 24.3 ± 3.5 °C. This stage occurs after the inoculation and before the mycelium fully colonizes the substrate. The temperature during incubation is crucial, as it affects the mycelium growth rate and vigor [22,23]. To simplify the exposure of the sample surfaces to air, the incubation period was divided into three phases: the first phase was the horizontal growth of molds; the second phase was the vertical growth of molds; and the third phase was the growth of mold without a mold. In the vertical growth position, the samples were reversed around 180 degrees to compensate for the loss of moisture in the top layer. This was undertaken to counteract the moisture decrease in the top layer due to the gravitational sinking of the moisture [17,24]. During incubation, it is important to monitor the growth of the mycelium to ensure its healthy development.

### 2.4. Dry Method

The drying process of the samples was performed in a laboratory oven to prevent mycelial growth and remove excess moisture. Drying time is 2 to 5 h, and the temperature range is 60 °C to 70 °C. When the mycelium growth stops by thermal treatment, its filaments are no longer supported by internal hydrostatic pressure, and for this reason, they appear to be flattened [13].

**Figure 2:**
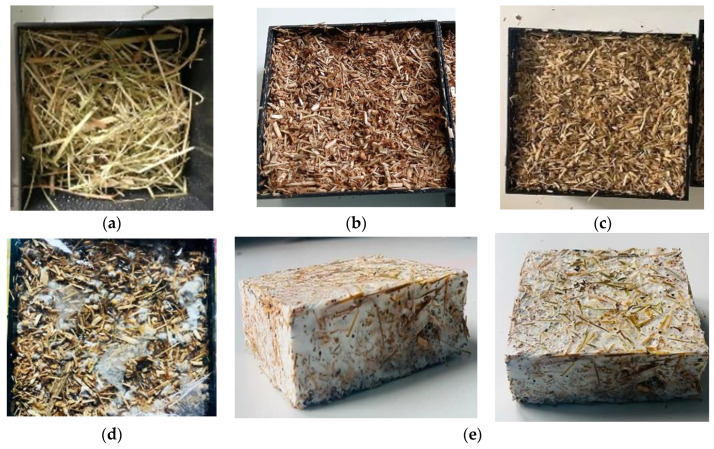
Stages of the mycelium-based composites: (**a**) substrate preparation; (**b**) inoculation *G. lucidum*; (**c**) inoculation *T. versicolor*; (**d**) incubation; and (**e**) dry method.

In Figure 2 shows stages of the mycelium-based composites: substrate preparation, inoculation *G. lucidum*/*T. versicolor*, incubation, and dry method.

Two sets of samples were prepared in heat-resistant molds (100 mm × 100 mm × 40 mm) with a formulation of 90% matrix and 10% fungal spores in the composite. A set of 5 samples used *G. lucidum* species and a set of 2 samples used *T. versicolor*. The initial thickness at the beginning was 40 mm, but after the drying process, the final thickness of the experimental sample reached 30 mm due to the removal of excess moisture.

### 2.5. Testing and Assessing Sound Absorption of Mycelium-Based Composites

Sound absorption is one method of acoustic treatment in which the energy of a sound wave is converted into low-grade heat, reducing the strength of reflected sound [18]. This reduces the amount of sound perceived as well as the effects of acoustic discomfort. Sound absorptive materials have many different applications within architectural, studio, automotive, and industrial acoustics. They can be used as interior lining in vehicles, aircraft, ducts, industrial equipment, and buildings/interiors. These materials are notably used within performance spaces to control unwanted echo, work environments to quiet the reverberant field, and restaurants to improve users’ communication. A measurement of a material’s sound absorption is called the sound absorption coefficient, which is the ratio of energy absorbed to the incident energy. The higher the sound absorption coefficient, the more absorptive the material.

Measurements for the materials were determined using an impedance tube according to SR EN ISO 10534-2 standard [25] based on the transfer function method. The testing equipment is part of a complete acoustic material analysis system featuring a Brüel and Kjaer Type 4206 a medium impedance tube (Brüel&Kjær, Nærum, Denmark), two 4187 Brüel and Kjaer microphones (Brüel&Kjær, Nærum, Denmark), an Analyser PULSE 3560-B030 (Brüel&Kjær, Nærum, Denmark) acoustic signal generator, a 2716 Brüel and Kjaer signal amplifier (Brüel&Kjær, Nærum, Denmark), and a PC for recording and processing results, connected with the Brüel and Kjaer PULSE interface.

The specific environmental conditions under which the measurements were performed to determine the absorption coefficient of mycelium-based composites are as follows: atmospheric pressure = 1000 hPa; temperature = 25.6 °C; relative humidity = 46%; speed of sound = 346.5 m/s; air density = 1.164 kg/m^3^; characteristic air impedance = 403.3 Pa. The frequency range considered was 50–3150 Hz.

For each studied recipe, three boards were made from which we cut a test specimen. We tested three test specimens from three different boards from the same recipe. From each recipe, only those panels in which the mycelium had developed adequately were chosen to be studied from an acoustic point of view.

**Figure 3:**
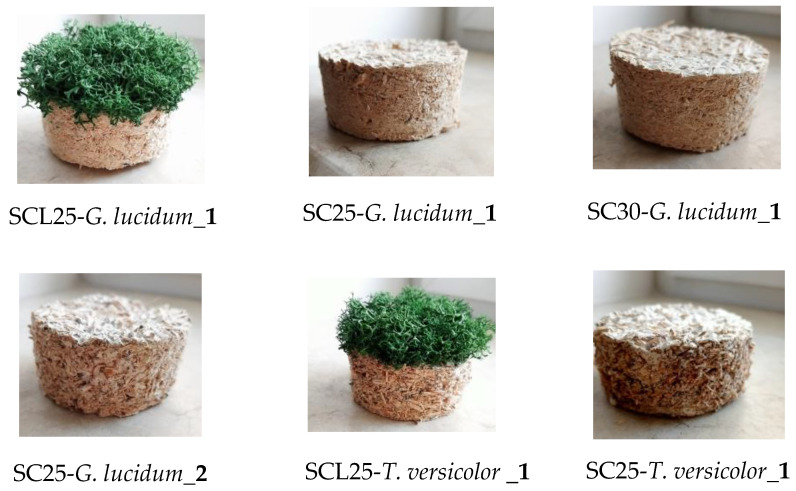
The sample set for determining the sound absorption coefficient.

Samples were selected and then cut using a circular shape with a diameter of 63.5 mm (Figure 3) and different widths (25 and 30 mm). Lichens were added to the surfaces of two samples containing mycelium *G. lucidum* and *T. versicolor* to improve the aspect and to observe their influence on sound absorption. The coding of the samples used to determine the acoustic absorption coefficient is presented in Table 3.

Two samples with different degrees of mycelial development were selected from the first set of samples prepared with *Ganoderma lucidum*. The mycelium of sample SC25-*G. lucidum_***1** is more developed, resulting in a denser composite material, while the mycelium of sample SC25-*G. lucidum_***2** is less developed, resulting in a lower sample density and higher open porosity.

**Figure 4:**
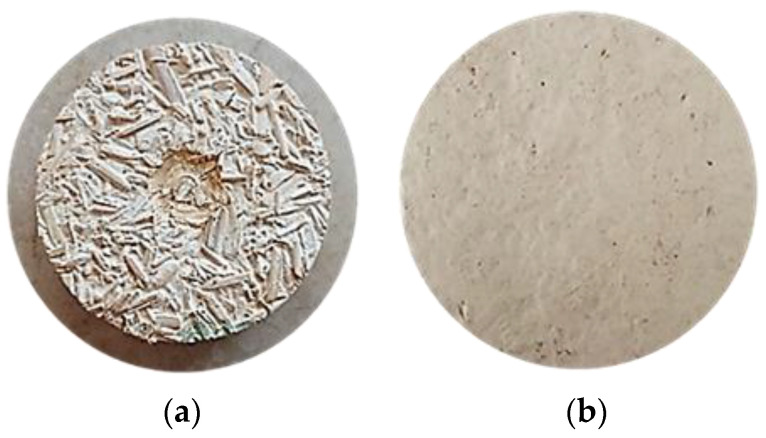
The surface area of the sample SC25-*G. lucidum*_1: (**a**) top in the mold (face) and (**b**) bottom in the mold (back face).

The sound absorption coefficient of samples SC25-*G. lucidum_***1** and SC30-*G. lucidum_***1** was measured on both surfaces of the composite (top and bottom in the mold) since they were found to have different roughness via visual analysis (Figure 4). The contact surface is an important parameter for sound absorption, so the roughness of the contact surface is also taken into account.

## 3. Results and Discussion

### 3.1. Testing and Assessing Sound Absorption of Mycelium-Based Composites

The selection of fungal species that efficiently produce bio-composite materials was based on specific criteria, such as mycelial density, growth rate, cost of growth medium (substrate), non-toxicity, and mycelial structure. These factors were taken into consideration to ensure that the most effective and sustainable materials were produced. 

To observe the development of the mycelium, a visual inspection was carried out, and the best samples were selected to prepare for the determination of the sound absorption coefficient. The mycelium formed during the incubation process appears in the structure of the material as a branching network of thin filaments called hyphae. They grow around the raw material and bind the particles together, acting like adhesives in solid composites. The way mycelium interacts with the provided substrate differs significantly between fungal species, depending on their morphological, biochemical, and physicochemical structure.

Although there are limited studies in this area on how material properties change, they point to important connections between the genetic nature of the species used and the mechanical properties of the cell wall composition. For example, *T. versicolor* materials have higher compressive strength and stiffness compared to *G. lucidum* [13]. 

**Figure 5:**
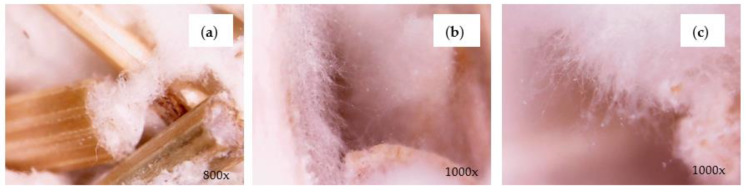
Microscopic representation of the mycelium of SC-*G. lucidum*_**1**: (**a**) mycelial network; (**b**); and (**c**) micro-filaments (hyphae).

**Figure 6:**
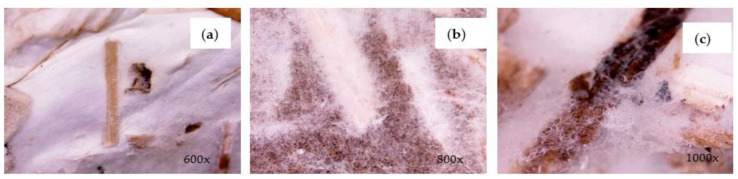
Microscopic images of the mycelium of SC-*T. versicolor* _**1**: (**a**) fungal membrane; (**b**) branched network of hyphae; (**c**) mycelial micro-filaments.

When visually analyzed with a digital microscope electronic magnifying glass, *G. lucidum* (Figure 5) mycelium samples had a fluffier and denser morphology, similar to cotton fibers, compared to *T. versicolor* (Figure 6), in which the mycelium had a stronger material structure, showing an airy and irregular condition. After 16 days of growth (incubation), a uniform fungal film formed on the sample surface. Aspects have also been identified in the literature [5,8].

When visually analyzed with a digital microscope electronic magnifier, it was observed that the self-grown fibrous film covers all the area of the feeding substrate after the specific growing period. As may be expected [17,26], the growing period was identical for both mycelia species, which belong to the same group of white rot fungi so they can excrete similar enzymes, and the substrates, in both cases, were rich in polysaccharides (organic compounds found in the composition of spent coffee grounds). Although, in both cases, a fibrous fungal membrane was formed on the external surface, their individual morphologies under the microscope show differences at the end of the growth stage.

For samples with *G. lucidum* mycelium (Figure 5), the density of micro-filaments was clearly increased with growth time, reaching a compact porous structure after about 20 days, similar to cotton fibers. It can also be seen that the micro-filaments have a tube-like and thread-like structure, and the average width was about 0.8 μm.

Compared to *T. versicolor* (Figure 6), the micro-filaments have a tangled short tube type structure; thus, the formed mycelial network contains air pockets and irregularities. After the drying process, darker areas appeared on the fungal membrane (brown spots), identified exclusively at the bottom. The specialized literature attributed the “browning” of the fungal membrane during the drying stage to the Maillard reaction of sugars and proteins [27].

**Figure 7:**
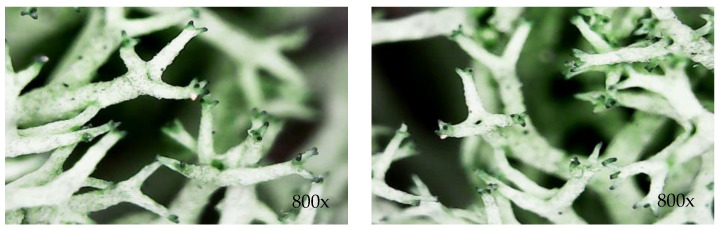
Microscopic images of lichens.

At the microscopic level, it was observed that the morphology of lichens (Figure 7) is formed by branched and tubular networks of filaments with air spaces which favor a more efficient absorption of sound waves. However, due to a very large opening of the lichen structure, the internal friction process is low, which means that the absorption improvement is not very high.

### 3.2. Sound Absorption of the Mycelium-Based Composites

Sound absorption performance can be improved due to sound energy reduction by interconnectivity, number, size, and type [28,29]. The absorption capacity of the experimental materials is influenced by certain parameters that were analyzed according to the data obtained: the type of mycelium, the thickness of the material, the presence of lichens, and the contact surface. The results of the impedance tube for each sample were recorded and graphed.

The ability of a material to absorb sound is mostly determined by its interior structure. One of the most essential elements investigated in this study was the specific type of mycelium used in the manufacturing of sound-absorbing materials.

**Figure 8:**
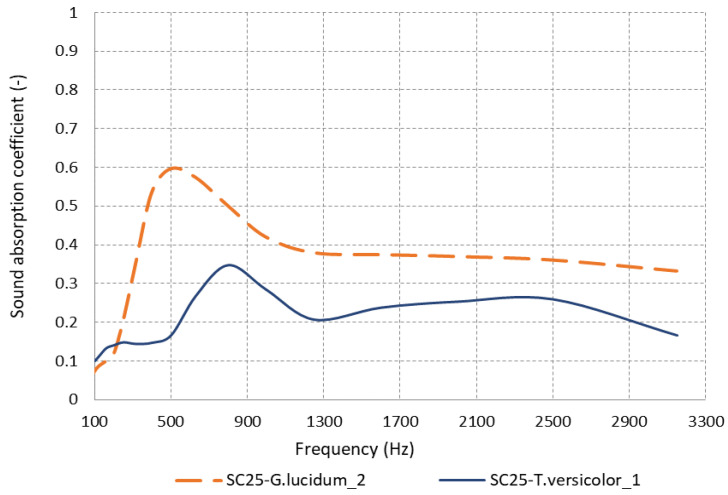
Sound absorption coefficient variation according to mycelium type.

Figure 8 provides a clear representation of the mycelium type’s impact on the materials’ absorption performance. The graphical representation of the data reveals that frequencies above 300 Hz show an enhanced absorption capacity of SC25-*G. lucidum*_**2**, with the maximum absorption coefficient value being 0.5965 at 500 Hz. When compared to the substrate in which the mycelium *of T. versicolor* was used, it was observed that the material with *G. lucidum* mycelium exhibited superior acoustic wave absorption properties at frequencies exceeding 500 Hz. The augmented sound absorption of the *G. lucidum* material is primarily attributed to its porosity, which results from the more-open pores created in the cellular matrix of the binder and the substrate granules. This is illustrated in Figure 5. Theoretically, the acoustics absorbing properties of a porous material are mainly influenced by properties of the pore; large and interconnected pores favor sound absorption at low frequencies [30]. Conversely, the SC25-*T. versicolor*_**1** sample shows a more compact and irregular mycelial network, as depicted in Figure 6, and a fungal membrane has formed on the contact surface, leading to a reduction in sound wave absorption. The porous material provides transmission paths for sound waves and favors sound absorption at lower frequencies [31]. 

**Figure 9:**
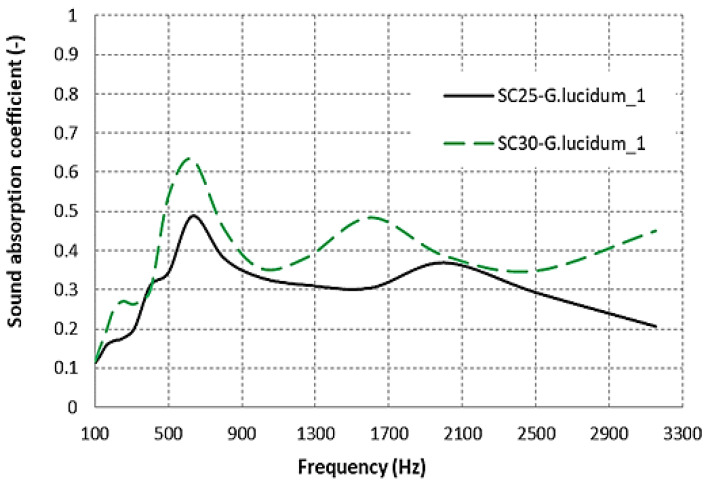
Sound absorption coefficient variation according to the mycelium thickness of the composite.

It has been established in the literature that material thickness is a relevant factor affecting acoustic performance. As the material thickness increases, the sound absorption coefficient improves, particularly at low frequencies but also at higher frequencies. Figure 9 presents a graphical representation of the dependency of the sound absorption coefficient on material thickness for sound-absorbing materials derived from coffee grounds and agricultural waste. It can be observed that the thickness of the materials SC25-*G. lucidum*_**1** and SC30-*G. lucidum*_1 is 25 mm and 30 mm, respectively. More research on the influence of thickness on acoustics absorption concluded that with increasing thickness, the sound absorption coefficient increases at low frequencies [32,33].

When examining the material with a thickness exceeding 30 mm (SC30-*G. lucidum*_**1**), it becomes apparent that its sound absorption coefficient exhibits a higher degree of oscillation across nearly the entire frequency spectrum, with its maximum point reaching 0.6332. Additionally, there is a noticeable increase in the absorption properties of the thicker material at frequencies below 300 Hz. In comparison to the 25 mm thick material (SC25-*G. lucidum*_**1**), the absorption coefficient variation decreases consistently with increasing frequency up to 1300 Hz, remaining approximately constant around 0.38 thereafter, excepting the frequency range of 400–900 Hz. As a comparative analysis, this aspect was also identified for the fine cardboard sample (FCL), which showed the best absorption, though none of the samples showed very high absorption in the low-frequency interval (50–500 Hz). It was noted that sound absorption results shown do not include the effect of an air gap behind the material. The introduction of an air cavity between the material and the rigid backing surface can increase the sound absorption performance at low frequencies [34].

This study also focuses on the impact of lichen existence on sound absorption coefficients. Lichen is a natural material that has been found to have sound absorption properties which can be exploited for various applications, such as soundproofing and acoustic enhancement [20]. Through examining the relationship between the two variables, it is hoped that a better understanding of the role of lichen in the acoustic environment can be gained. The data collected will be analyzed using statistical methods to determine the significance of the results. The findings of this study will be useful in developing methods to control noise pollution and in the design of sound-absorbing materials that utilize the natural properties of lichen.

**Figure 10:**
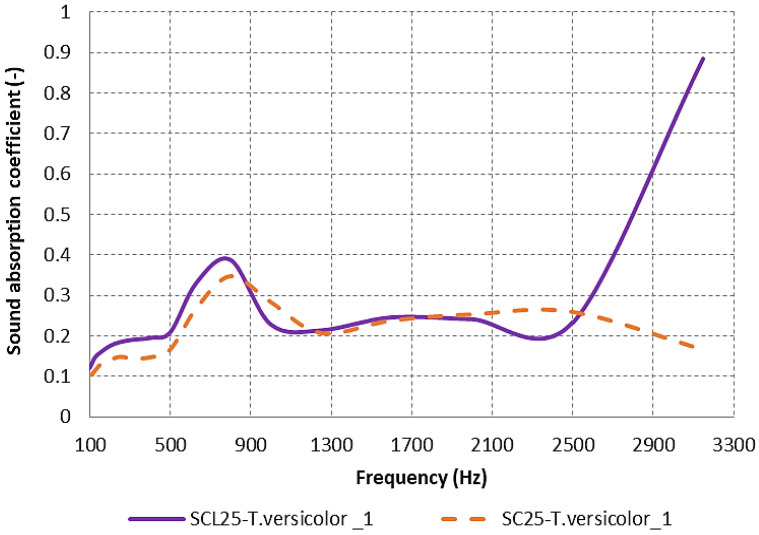
Sound absorption coefficient variation according to the presence of lichens for *T. versicolor* mycelium composite.

According to the graphical representation in Figure 10, the material with a lichen-covered exterior surface, SCL25-*T. versicolor_***1**, shows much better sound absorption properties at high frequencies above 2500 Hz, with a maximum point of 0.8843 being observed. Also, an increased absorption coefficient at high frequencies was observed in a study by Natalie Walter, where the samples with shredded cardboard content in the recipe and *Pleurotus ostreatus* mycelium (SHC) had the highest sound absorption (above the point of 0.9) in the interval 2000–6000 Hz [34].

After conducting measurements and analyzing the resulting data, it was discovered that the existence of lichens on the exterior of SCL25-*T. versicolor*_**1** had a positive impact on its sound absorption capabilities. The maximum value was observed within the 500–900 Hz frequency range. On the other hand, the denser surface of the SC25-*T. versicolor*_**1** sample led to lower sound absorption, with greater values occurring in the 600–800 Hz frequency range, as indicated in Figure 10.

**Figure 11:**
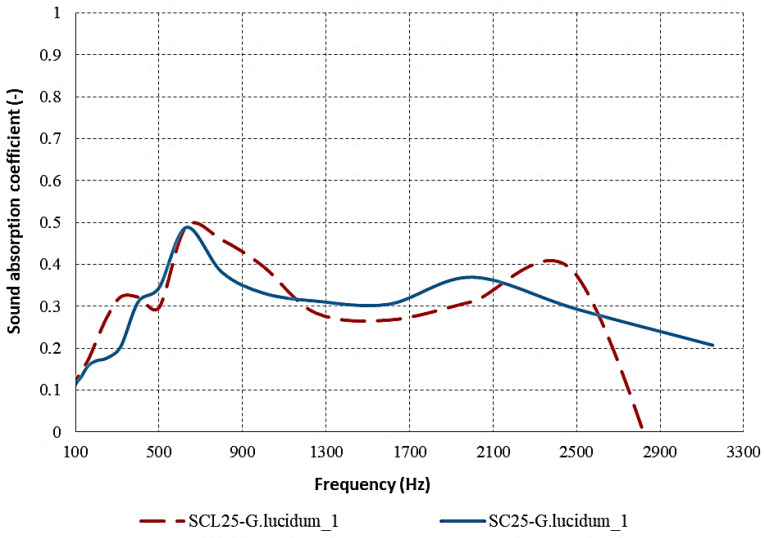
Sound absorption coefficient variation according to the presence of lichens for *G. lucidum* mycelium composite.

To analyze the absorption capacity of sample SCL25-*G. lucidum*_**1** in comparison to reference materials, the presence of lichens was also taken into account. Figure 11 reveals that the SC25-*G. lucidum*_**1** sample displays notable absorption properties over a wide frequency range due to the presence of vacant spaces in its material structure, which allows for better absorption of sound waves. Additionally, the absorption coefficient for frequencies ranging between 500 and 900 (0.4875) is at its maximum for the material with a lichen surface; however, the acoustic properties gradually decrease at medium frequencies. Conversely, the SC25-*G. lucidum*_**1** material does not exhibit favorable absorption capacity due to the development of a more densely packed mycelial network and a lack of porosity, which were intended for acoustic and thermal insulation purposes.

The texture of the contact surface is a parameter that influences sound absorption due to factors such as the diffuse surface of the material reflecting sound waves in several directions or surfaces with open pores so that part of the sound energy is absorbed [35]. To analyze the influence of this parameter, three material samples with *G. lucidum* mycelium were chosen at 25 mm and 30 mm width, respectively, and one sample with the outer surface in contact with lichens to improve the appearance and absorption.

**Figure 12:**
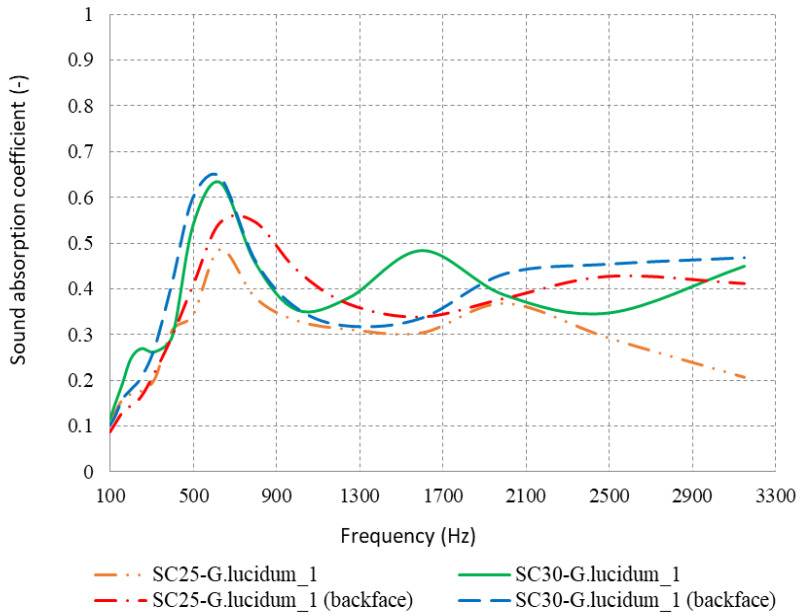
Sound absorption coefficient variation depending on the contact surface.

In Figure 12, the variation of the absorption coefficient for the upper and lower surface of the material sample analyzed (SC25-*G. lucidum*_**1**) is represented graphically. An improvement of the sound absorption in the low-frequency range (500–800 Hz) is noticed because the sound wave encounters the bottom surface of the material, which is flat and has open pores. The analysis samples in Figure 4 were debitage from the reference material SC-*G. lucidum*_**1**; thus, the mycelium formed as an absorbing membrane-like coating on the lower surface with the role of absorbing sound energy at low frequencies. Compared to the upper surface of the material, the raw materials used in the composition of the substrate are also observed, which prevents the absorption of sound waves, some of which are reflected.

In the case of the 30 mm section, this parameter increases the absorption capacity of the material and is also influenced by the lower contact surface. According to the graph in Figure 12, the maximum point of the sound absorption coefficient (0.6445) at the frequency of 600 Hz is reached by the material SC30-*G. lucidum*_**1**, for which the sound waves were attenuated by the flat, open-pored bottom surface. Therefore, the increase in material thickness and the change in contact surface significantly influence the sound absorption performance for the SC30-*G. lucidum*_**1** sample, with an ascending variation observed at higher frequencies (after 1700 Hz) compared to the rest of the analyzed samples, where the acoustic properties were visible at low frequencies.

## 4. Conclusions

In recent years, as a result of European Union regulations dedicated to avoiding and tackling pollution, the scientific community has focused on the possibility of employing waste to develop composite materials that have multiple advantages.

Various raw materials were evaluated based on multiple factors such as their market availability, cost, nutritional value, degradability, and structural properties. Among these raw materials, coffee grounds were selected as the primary nutrient source for the growth and advancement of the mycelial network due to their chemical composition, which includes carbohydrates, proteins, minerals, and caffeine.

A substrate composition has been established to obtain the proper experimental materials. The mixture was developed out of a variety of raw materials, including coffee grounds, coffee husks, hay straw, hemp straw, and a selection of cereals. The mycelium demanded for binding these essential elements was developed by cultivating spores from two fungi, *Ganoderma lucidum*, and *Trametes versicolor*.

The key factors impacting the absorption coefficient were investigated, including the kind of mycelium employed, the material thickness, the presence of lichens, and the contact surface. It was observed that the application of lichens to the surface of sound-absorbing material led to a notable improvement in both the visual and absorption properties of SCL25-*T.versicolor*_1, particularly at higher frequencies beyond 2500 Hz. Furthermore, the contact surface of the material was found to be a crucial factor in determining the degree to which sound waves could be attenuated, with greater thickness in the sound-absorbing layer correlating with improved attenuation. Based on comparative analysis, it was determined that the sample SC30-*G. lucidum*_1 exhibited exceptional sound absorption properties at the lower surface.

In conclusion, the material SC30-*G. lucidum*_1 has shown promising sound-absorbing properties at low frequencies, highlighting its potential in addressing the challenge of creating effective sound-absorbing materials for various applications.

## Figures and Tables

**Table 2:** Apparent density of the substrate materials.

Substrate Material	Spent Coffee Grounds	Coffee Chaff	Hay Straw	Hemp Dust	Cereal Mixture
Apparent density (g/cm^3^)	0.664	0.071	0.018	0.15	0.675

**Table 3:** Sample codification.

Code	Lichens	Thickness (mm)	Fungal Species	Experimental Set
SCL25-*G. lucidum*_**1**	Yes	25	*Ganoderma lucidum*	1
SC25-*G. lucidum*_**1**	No	25		1
SC30-*G. lucidum*_**1**	No	30		1
SC25-*G. lucidum*_**1**	No	25		2
SCL25-*T. versicolor*_**1**	Yes	25	*Trametes versicolor*	1
SC25-*T. versicolor*_**1**	No	25		1

## Data Availability

Data are contained within the article.

## Abbreviations

MBCs	Mycelium-based composites
SHC	Shredded cardboard content
FCL	Fine cardboard sample
